# Deregulation of the Histone Lysine-Specific Demethylase 1 Is Involved in Human Hepatocellular Carcinoma

**DOI:** 10.3390/biom9120810

**Published:** 2019-12-01

**Authors:** Sangchul Kim, Amina Bolatkan, Syuzo Kaneko, Noriko Ikawa, Ken Asada, Masaaki Komatsu, Shinya Hayami, Hidenori Ojima, Nobutsugu Abe, Hiroki Yamaue, Ryuji Hamamoto

**Affiliations:** 1 Division of Molecular Modification and Cancer Biology, National Cancer Center Research Institute, 5-1-1 Tsukiji, Chuo-ku, Tokyo 104-0045, Japan; kim3.com@gmail.com (S.K.); abolatka@ncc.go.jp (A.B.); nikawa@ncc.go.jp (N.I.); ken.asada@riken.jp (K.A.); maskomat@ncc.go.jp (M.K.); 2 Department of Gastroenterological and General Surgery, Kyorin University School of Medicine, 6-20-2 Shinkawa, Mitaka, Tokyo 181-8611, Japan; abenbtg@ks.kyorin-u.ac.jp; 3 Cancer Translational Research Team, RIKEN Center for Advanced Intelligence Project, 1-4-1 Nihonbashi, Chuo-ku, Tokyo 103-0027, Japan; 4 Second Department of Surgery, School of Medicine, Wakayama Medical University, 811-1 Kimiidera, Wakayama 641-8510, Japan; shin-8@wakayama-med.ac.jp (S.H.); yamaue-h@wakayama-med.ac.jp (H.Y.); 5 Department of Pathology, Keio University School of Medicine, 35 Shinanomachi, Shinjuku-ku, Tokyo 160-8582, Japan; hojima@a3.keio.jp

**Keywords:** hepatocellular carcinoma, LSD1, histone demethylase

## Abstract

Hepatocellular carcinoma (HCC) is the most common type of primary liver cancer and is a leading cause of cancer-related death worldwide. Given that the standard-of-care for advanced liver cancer is limited, there is an urgent need to develop a novel molecular targeted therapy to improve therapeutic outcomes for HCC. In order to tackle this issue, we conducted functional analysis of the histone lysine-specific demethylase (LSD1) to explore the possibility that this enzyme acts as a therapeutic target in HCC. According to immunohistochemical analysis, 232 of 303 (77%) HCC cases showed positive staining of LSD1 protein, and its expression was correlated with several clinicopathological characteristics, such as female gender, AFP (alpha-fetoprotein) levels, and HCV (hepatitis C virus) infectious. The survival curves for HCC using the Kaplan–Meier method and the log-rank test indicate that positive LSD1 protein expression was significantly associated with decreased rates of overall survival (OS) and disease-free survival (DFS); the multivariate analysis indicates that LSD1 expression was an independent prognostic factor for both OS and DFS in patients with HCC. In addition, knockout of LSD1 using the CRISPR/Cas9 system showed a significantly lower number of colony formation units (CFUs) and growth rate in both SNU-423 and SNU-475 HCC cell lines compared to the corresponding control cells. Moreover, LSD1 knockout decreased cells in S phase of SNU-423 and SNU-475 cells with increased levels of H3K4me1/2 and H3K9me1/2. Finally, we identified the signaling pathways regulated by LSD1 in HCC, including the retinoic acid (RA) pathway. Our findings imply that deregulation of LSD1 can be involved in HCC; further studies may explore the usefulness of LSD1 as a therapeutic target of HCC.

## 1. Introduction

Hepatocellular carcinoma (HCC) is one of the most common cancer types and the second most common cause of death from cancer worldwide [1]. Many studies have revealed that hepatocarcinogenesis has the following multistep process: Activation of oncogenes and inactivation of tumor suppressor genes due to genetic and/or epigenetic events [2,3,4]. However, the precise molecular mechanisms relevant to HCC development are still uncertain. Since biochemical and genetic mechanisms involved in the development of cancers differ based on cancer type [5], the treatment of each cancer type often requires specified agents; this implies the importance of detailed functional analysis of each cancer type.

Epigenetic regulators have recently been implicated as key factors in many pathways related to cancer development and progression, including cell cycle regulation [6,7,8,9,10], invasiveness [11], signaling pathways [12], chemo-resistance [13], and immune evasion [14], in addition to genetic alterations. The three main systems of epigenetic regulation are DNA methylation of gene regulatory regions, covalent modifications of histones, such as methylation and acetylation, and non-coding RNAs. Among them, histone methylation is dynamically regulated by two different types of enzymes, called histone methyltransferases and histone demethylases. So far, approximately 50 different histone lysine methyltransferases (HKMTs) [15], 10 histone arginine methyltransferases (HRMTs) [15], and 30 histone demethylases (HDMs) [16] have been identified, but biological and physiological functions of these enzymes are still not fully clarified. On the other hand, on the basis of frequent overexpression and/or somatic mutations in a variety of cancer types, extensive efforts for the development of drugs targeting histone methyltransferases and histone demethylases have been progressed for the past several years [6,8,9,10,17,18,19,20,21,22]. Hence, in order to find histone methyltransferases and demethylases involved in human HCC, we screened a number of these enzymes in clinical tissues by expression profile analysis, and found transactivation of histone lysine-specific demethylase 1 (LSD1) in HCC tissues.

LSD1, also known as KDM1A, is a histone demethylase that does not belong to the JmjC family, and affects gene expression by selectively demethylating H3K4me2/me1 and H3K9me2/me1 [23,24]. Dysregulation of LSD1 is detected in various types of human cancer and correlates with poor outcome in cancer patients [25,26,27]. Importantly, LSD1-specific inhibitors have recently been developed [28], and in particular, a Phase IIa clinical trial of the novel LSD1 inhibitor Iadademstat (ORY-1001) in combination with Azacitidine has just started for acute myeloid leukemia (AML) (ALICE study); also a Phase IIa clinical trial of Iadademstat in combination with platinum-etoposide chemotherapy in patients with relapsed, extensive-stage disease small cell lung cancer (SCLC) (CLEPSIDRA study) has started. These imply that some LSD1 inhibitor is possible to use as an anti-cancer drug on the clinical spot in the future. On the other hand, although correlation between overexpression of LSD1 and HCC prognosis has been reported previously, the further precise molecular mechanisms between LSD1 and HCC still remain unclear.

In the present study, we aimed to investigate the clinical importance of LSD1 in HCC using a large number of clinical tissues, and clarified detailed molecular mechanisms based on functional analysis.

## 2. Materials and Methods

### 2.1. Patients and Tissue Samples

The retrospective study examined data of 422 patients who were diagnosed as primary hepatocellular carcinoma and performed curative liver resection at Wakayama Medical University Hospital (WMUH), Wakayama, Japan, from February 2000 to September 2014. Informed consent was obtained from all patients in accordance with the guidelines of the Ethical Committee on Human Research of WMUH (approval number: 871) [29]. The exclusion criteria were as follows: Patients who had received transcatheter arterial embolization (TAE) preoperatively; those who had undergone liver resection for liver metastasis; those who had died of other diseases; those who had undergone noncurative resection. No patients who had received preoperative chemotherapy or radiotherapy were included. Finally, a total of 303 patients were enrolled in the study. Diagnosis of HCC was identified by World Health Organization criteria; the Child-Pugh scoring system was used for assessing hepatic impairment. Tumor category and stage were determined according to the 8th edition of the tumor-node-metastasis (TNM) classification of the Union for International Cancer Control (UICC).

### 2.2. Immunohistochemistry

The expression patterns of LSD1 in human liver tissues were examined by immunohistochemistry as described previously [30,31,32,33]. Briefly, slides of paraffin-embedded liver tumor specimens were processed under high pressure (125 °C, 30 s) in antigen-retrieval solution, high pH 9 (S2367, Dako, Carpinteria, CA, USA), treated with peroxidase blocking regent, and then treated with protein blocking regent (K130, X0909, Dako). Tissue sections were incubated with the rabbit anti-LSD1 polyclonal antibody (ab17721, abcam, Cambridge, UK; dilution used in IHC: 1:200), followed by HRP-conjugated secondary antibody (Dako). Antigen was visualized with substrate chromogen (Dako liquid DAB chromogen; Dako). Finally, tissue specimens were stained with Mayer hematoxylin (Hematoxylin QS; Vector Laboratories, Burlingame, CA, USA) for 20 s to discriminate the nucleus from the cytoplasm.

### 2.3. Evaluation of Immunohistochemistry

LSD1 was immunohistochemically analyzed by two independent experienced pathologists who were blinded to the clinical data. Immunoreactivities of LSD1 were defined as follows: 0+, no nuclear staining; 1+, nuclear staining, equivalent to the intensity of the normal hepatocyte epithelium (NHE); 2+, nuclear staining, higher than the intensity of the NHE within the same section. The IHC score of 2+ of LSD1 was defined as positive for expression.

### 2.4. Cell Lines

The human hepatocellular carcinoma cell lines SNU-423 and SNU-475 were purchased from the American Type Culture Collection (ATCC; Manassas, VA, USA), and tested and authenticated by DNA profiling for polymorphic short tandem repeat (STR) markers (Supplementary Table S1). SNU-423 and SNU-475 cells were cultured in monolayers in RPMI-1640 supplemented with 10% FBS and antibiotics. All cells were maintained at 37 °C in humid air with 5% carbon dioxide (CO_2_) and 95% air. 

### 2.5. Plasmid DNA Constructs

The lentiviral packaging plasmids, pMD2.G and psPAX2 were obtained from Addgene (#12259 and #12260, Watertown, MA, USA). To generate inducible Cas9 nuclease-expression cell lines, we purchased Edit-R Inducible Lentiviral Plasmid (#CAS11229, Dharmacon, Lafayette, CO, USA). Two individual sgRNAs to target *LSD1* gene—sgRNA1, 5′-TATAAGGTGCTTCTAATTGT-3′ and sgRNA2, 5′-AGAGCCGACTTCCTCATGAC-3′—were designed and cloned into pLKO.1-puro U6 sgRNA BfuAI large stuffer (#52628, Addgene). All plasmids were verified by Sanger sequencing.

### 2.6. Lentiviral Transduction

To produce lentiviruses, viral vector and packaging plasmids were cotransfected into the 293T cells with Lipofectamine^®^ 2000 (Thermo Fisher Scientific, Waltham, MA, USA) according to the manufacturer’s instructions. After 48 h, cell culture medium containing lentiviruses was collected and filtered through a 0.45-μm filter. Lentiviral transduction of SNU-423 and SNU-475 cells was carried out in the absence of polybrene.

### 2.7. Generation of LSD1 Knockout Cell Lines Using the CRISPR/Cas9 Gene Editing System

Lentiviruses were prepared as described above. Stable cell clones were then selected in the presence of Blasticidin S (10 μg/mL) and Puromycin (2 μg/mL). Knockout of *LSD1* gene was induced by adding doxycycline (Dox) (1 μg/mL) for 24 h. To reduce off-target of gene editing, we replaced medium without Dox.

### 2.8. Western Blotting Assays

The SNU-423 and SNU-475 cells were lysed with denaturing SDS-PAGE sample buffer using standard methods. Protein lysates were separated on a 10% SDS-PAGE gel and transferred to the nitrocellulose membranes. The membranes were blocked with 5% skimmed non-fat milk for 1 h at room temperature, and then the membranes were incubated with anti-LSD1 (ab17721, abcam; dilution used in WB: 1:2000), anti-mono-methyl Histone H3-K4 (H3K4me1, ab8895, abcam; dilution used in WB: 1:2000), anti-di-methyl Histone H3-K4 (H3K4me2, #9725, Cell Signaling Technologies, Danvers, MA, USA; dilution used in WB: 1:1000), anti-mono-methyl Histone H3-K9 (H3K9me1, #14186, Cell Signaling Technologies; dilution used in WB: 1:1000), anti-di-methyl Histone H3-K9 (H3K9me2, #4658, Cell Signaling Technologies; dilution used in WB: 1:1000), anti-Histone H3 (#4499, Cell Signaling Technologies; dilution used in WB: 1:2000), and anti-α-Tubulin (DM1A, EMD Millipore, Burlington, MO, USA; dilution used in WB: 1:1000) antibodies at 4 °C overnight. After primary antibody incubation, the membranes were incubated with HRP-conjugated secondary antibody at room temperature for 1 h. The signal was detected by ECL system (GE Healthcare, Chicago, IL, USA).

### 2.9. Colony Formation Assays

The infected cells were seeded in 6-well plates at density of 500 cells/well, and cultured at 37 °C. Medium was replaced every 3 days. After 14 days, the colonies were fixed with methanol and stained with 0.1% crystal violet. Visible colonies were manually counted. Triplicate wells were assessed for each treatment group.

### 2.10. Cell Proliferation Assays

The infected cells were seeded in 96-well plates at density of 1.0 × 10^3^ cells/well, and cultured for 96 h. Cell viability was measured by the Cell Counting Kit-8 (CCK-8) system (Dojindo Laboratory, Kumamoto, Japan) following the manufacturer’s protocol [8,19,32,33,34,35]. Briefly, CCK-8 solution (10 μL per 100 μL of medium in each well) was added at 0, 24, 48, 72, and 96 h post-treatment, the plates were then incubated at 37 °C for 1 h, and absorbance each well was read at 450 nm using a microplate reader.

### 2.11. Cell Cycle Assays

To assess the cell cycle, the infected cells were seeded into 6-well plates at density of 0.5 × 10^6^ cells/well, and cultured for 72 h post-treatment. Cells were incubated with EdU for 2 h before harvest, fixed in Click-iT^®^ fixative for 15 min, and then incubated in propidium iodide (PI) staining buffer (50 μg/mL PI, 200 μg/mL RNase A, 0.01% Triton-X, PBS) for 30 min in the dark and at room temperature. EdU was detected using the Click-iT^®^ Plus EdU Alexa Fluor^®^ 488 Flow Cytometry Assay Kit (C10632, Thermo Fisher Scientific) following the manufacturer’s instructions. Cell cycle distribution was analyzed by flow cytometry (Cell Analyzer EC800, SONY, Tokyo, Japan).

### 2.12. RNA-Seq

For library preparation, polyA+ RNA was enriched from total RNA using oligo(dT)-attached magnetic beads. After fragmentation of RNAs, first-strand cDNA was generated using random hexamer-primed reverse transcription, followed by a second-strand cDNA synthesis. The synthesized cDNA was subjected to end-repair and then was 3′ adenylated. Adapters were ligated to the ends of these 3′ adenylated cDNA fragments. After PCR amplification, PCR products were purified with Agencourt AMPure XP Beads (Beckman Coulter, Brea, CA, USA). DNA library was validated on the 2100 Bioanalyzer (Agilent Technologies, Santa Clara, CA, USA). Sequencing was performed on BGI-seq 500 (BGI, Shenzhen, Guangdong, China). The statistics of the sequencing data production are summarized in Supplementary Table S2. Reads from all sequencing experiments were deposited under accession number DRA009192.

### 2.13. Statistical Analysis

All statistical analyses were performed with EZR (Saitama Medical Center, Jichi Medical University; http://www.jichi.ac.jp/saitama-sct/SaitamaHP.files/statmedEN.html; [36]), which is a graphical user interface for R (The R Foundation for Statistical Computing, Vienna, Austria, version 2.13.0). More precisely, it is a modified version of R commander (Version 1.6-3) that was designed to add statistical functions frequently used in biostatistics. Associations between LSD1 expression and patient’s characteristics were determined using Fisher’s exact test for categorical variables or Mann–Whitney *U* test for continuous variables. Follow-up was considered from the time of surgery to the date of death or last contact. Overall survival (OS) was computed from the date of surgery to the date of death correlated with HCC. Disease free survival (DFS) was computed from the date of surgery to the date of any metastasis, including intrahepatic metastasis, local recurrence, lymph node metastasis, peritoneal dissemination, and all the other distant metastases. Patients alive at the end of the study period were censored at the date of last follow-up or the last date the patient was known to be alive, whichever was longer. OS and DFS were assessed using Kaplan–Meier estimates and comparisons were performed using the log-rank test. The hazard ratios (HR) derived from Cox’s proportional hazards model. Time-to-event results are reported with HR, 95% confidence interval (CI) for the HR, and the log-rank *p*-value. All *p*-values were two sided and *p*-values of 0.05 or less were considered statistically significant. To assess whether overexpression of LSD1 was independently associated with clinical outcome, variables that were associated with OS and DFS at the *p*-value 0.20 level were included in multivariate Cox regression models after a backwards conditional method, in which the variable with the highest *p*-value was removed one at a time until all variables left in the model were significant at the 0.05 level. 

## 3. Results

### 3.1. Association of LSD1 Expression with the Clinicopathological Features of HCC

**Figure 1:**
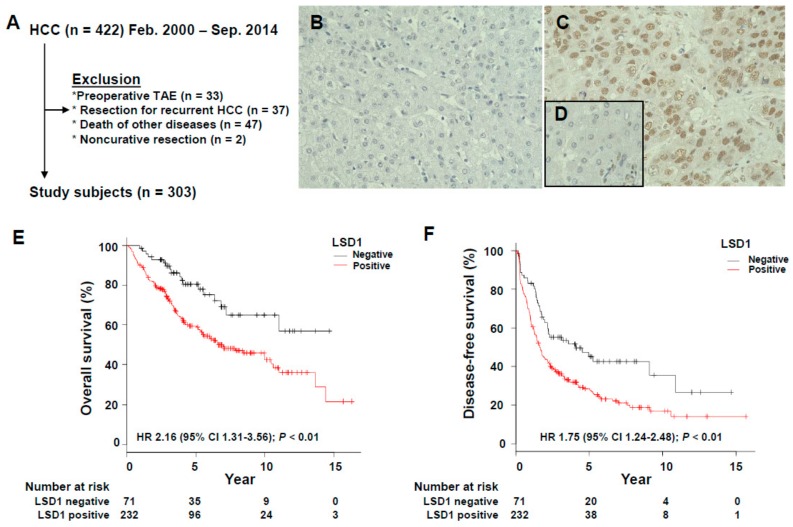
Immunohistochemical analysis of LSD1 in hepatocellular carcinoma (HCC) cases. (**A**) Flow chart of immunohistochemistry in this study. (**B**) Score 0 and (**C**) score 2+ for LSD1. (**D**) The nuclear LSD1 staining in normal hepatocyte epithelium was classified as score 1+. (**E**) Overall survival and (**F**) disease-free survival rate of HCC patients according to LSD1 expression.

The patient flow chart for the study is shown in Figure 1A. A total of 303 cases were enrolled in the study, and examples of LSD1 protein expression with IHC are shown in Figure 1B–D. LSD1 proteins were mainly localized in the nucleus both carcinomatous component and adjacent normal hepatocyte epithelium (NHE). Out of 303 HCC patients, 232 cases (77%) were positive, while 71 cases (23%) were negative. To further investigate the clinical significance of LSD1 protein expression in HCC, the associations between LSD1 staining results and clinicopathological features of HCC were statistically analyzed (Table 1). Subsequently, we found that LSD1 protein expression in HCC patients was significantly associated with female gender (*p* = 0.018, Fisher’s exact test), non-alcoholic abuse history (*p* = 0.0027), HCV (hepatitis C virus) infectious (*p* = 0.038), no infectious history (*p* = 0.013), AFP (alpha-fetoprotein) levels (*p* < 0.001), CA 19–9 levels (*p* = 0.017), hepatic vein invasion (*p* = 0.017) and fibrosis stage F4 (*p* = 0.0061).

### 3.2. Prognostic Significance of LSD1 Expression in HCC

The Kaplan–Meier plot was applied to evaluate the prognostic significance of LSD1 protein expression. Patients with positive LSD1 expression had both significantly lower OS and DFS rates than patients with negative expression (both *p* < 0.01, log-rank test; Figure 1E,F). To further assess the clinical significance of LSD1 expression as a prognostic predictor for HCC patients, the univariate analysis was performed with the 20 potential risk factors listed in Table 2. Positive LSD1 expression was significantly associated with decreased rates of OS and DFS (OS: HR, 2.16; 95% CI, 1.31–3.56. DFS: HR, 1.75; 95% CI, 1.24–2.4; Table 2). Given that Table 2 shows that not only LSD1 protein expression, but also the other factors such as AFP levels were associated with decreased rates of OS and DFS, the multivariate analysis was performed (Table 3 and Table 4). LSD1 expression was an independent prognostic factor for both overall and disease-free survival in patients with HCC.

### 3.3. Establishment of Dox-Inducible LSD1 Knockout HCC Cells Using the CRISPR/Cas9 System

**Figure 2:**
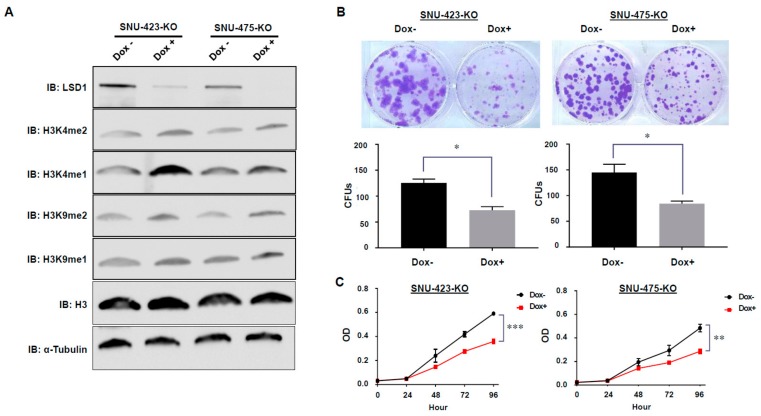
Effects of LSD1 knock-out on HCC cell viability in vitro. LSD1 knock-out SNU-423 cells and SNU-475cells were generated by the CRISPR/Cas9 system with two guide RNAs and were obtained by treatment with Dox. (**A**) Western blot analysis of LSD1, H3K4me2, H3K4me1, H3K9me2, H3K9me1, histone H3, and α-Tubulin in SNU-423-KO and SNU-475-KO cells. (**B**) Colony formation assays were conducted to determine the proliferation of infected HCC cells. All data are represented as mean ± s.d. * *p* < 0.05. (**C**) Cell proliferation assays were performed to determine the cell viability of infected HCC cells with the CCK-8 kit. All data are represented as mean ± s.d. ** *p* < 0.01; *** *p* < 0.001.

In order to evaluate functions of LSD1 in HCC cells, we established Dox-inducible LSD1 knockout SNU-423 (SNU-423-KO) and SNU-475 (SNU-475-KO) HCC cell lines using the CRISPR/Cas9 system. Subsequently, we conducted Western blot analysis to validate knockout efficiency of LSD1 protein in SNU-423-KO and SNU-475 KO cells, and confirmed that LSD1 was clearly knocked out in both cells after treatment with Dox (Figure 2A). In addition, we found up-regulation of H3K4me1/2 and H3K9me1/2 levels in both SNU-423-KO and SNU-475 KO cells after treatment with Dox (Figure 2A); this is concordant with former findings because H3K4me1/2 and H3K9me1/2 were reported as targets of LSD1-mediated demethylase activity [23,24]. Since we successfully established the Dox-inducible LSD1 knockout HCC cell lines (SNU-423-KO and SNU-475-KO), we further performed functional analysis of LSD1 in HCC using these cell lines.

### 3.4. Effects of LSD1 on Cell Growth and Cell Cycle in HCC Cells

**Figure 3:**
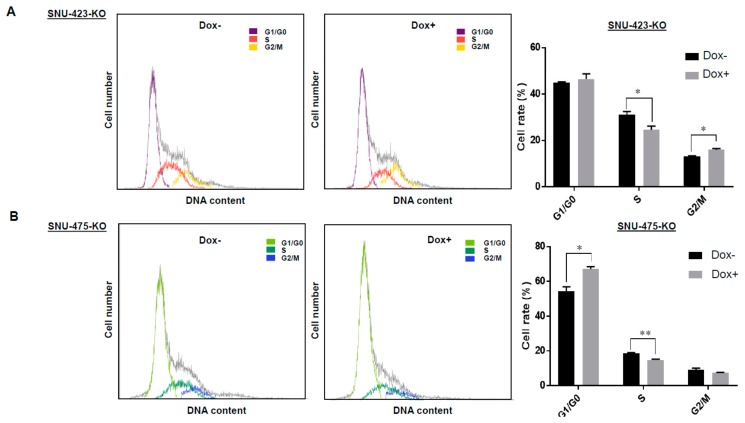
Effects of LSD1 knock-out on HCC cell cycle in vitro. LSD1 knock-out SNU-423 cells (**A**) and SNU-475 cells (**B**) generated by the CRISPR/Cas9 system with two guide RNAs were obtained by treatment with Dox. Flow cytometry was conducted to investigate the effects of LSD1 knockout on HCC cell cycle. LSD1 knockout decreased the number of cells at S phase in each cell lines. While it increased the number of cells at G2/M phase in SNU-423 cells, it increased the number of cells at G0/G1 phase in SNU-475 cells. All data are represented as mean ± s.d. * *p* < 0.01; ** *p* < 0.001.

To investigate whether LSD1 regulates HCC cell growth, colony formation assays were performed (Figure 2B). In this case, we firstly confirmed that Dox-treatment itself did not affect proliferation of SNU-423 and SNU-475 HCC cells (Supplementary Figure S1); we also validated that growth rates of these HCC cells were not changed after Cas9 expression. LSD1-knockout cells (Dox+) showed a significantly lower number of colony formation units (CFUs) in both cell lines (SNU-423-KO and SNU-475-KO), compared to the control cells (Dox-). Moreover, proliferation assays using CCK-8 showed that knockout of LSD1 significantly reduced the growth rate of SNU-423 and SNU-475 HCC cell lines (Figure 2C). These results imply that LSD1 appears to play a critical role in the growth regulation of HCC cells. Hence, we conducted cell cycle assays using flow cytometry to further explore the role of LSD1 in HCC growth regulation. As shown in Figure 3A,B, knockout of LSD1 decreased the number of cells at S phase in both SNU-423 and SNU-475 HCC cell lines, revealing that LSD1 is likely to be important for the G1/S phase transition in HCC cells. Additionally, given that the sub-G1 population of SNU-423 and SNU-475 HCC cell lines was not changed (Figure 3A,B), LSD1 knockout might not induce of these HCC cells. 

### 3.5. Transcriptome Analysis of LSD1 Knockout in HCC Cells

**Figure 4:**
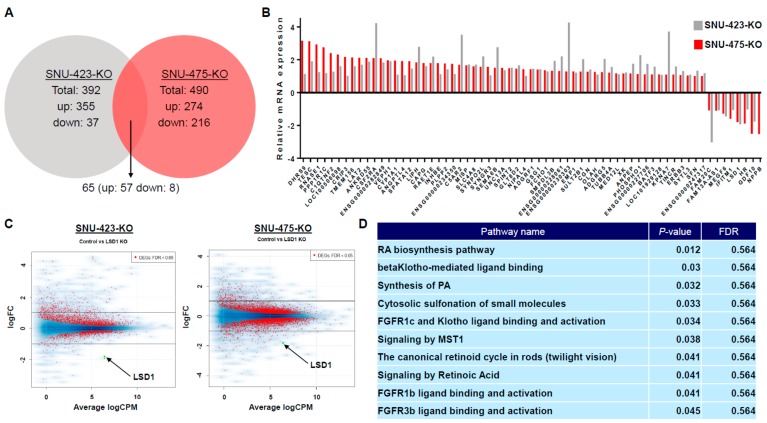
RNA-seq analysis using NNU-423-KO and SNU-475-KO cells. (**A**) Venn diagram of differentially expressed genes (DEGs) in SNU-423-KO and SNU475-KO cells. Sixty-five common significant differentially expressed genes were found; 57 were up-regulated after knocking out LSD1, and 8 were down-regulated, including LSD1. (**B**) Relative mRNA expression of DEGs in SNU-423-KO and SNU475-KO cells. (**C**) The MA plot of RNA-seq analysis. The distribution of DEGs in group comparison was shown after knocking out LSD1 in both SNU-423 and SNU-475 cell lines. (**D**) Gene set enrichment analysis and pathway analysis were performed using Reactome (https://reactome.org/). Sixty-four common significant differentially expressed genes, except for LSD1, in both SNU-423 and SNU-475 HCC cell lines were used for this analysis. FDR, false discovery rate.

Given that LSD1 was reported to be a transcriptional regulator through altering histone modification status [37,38], we hypothesized that LSD1 might control the growth of HCC cells via regulating its downstream genes. We next performed RNA-seq analysis to identify the differences of gene expression in SNU-423 and SNU-475 HCC cell lines after knockout of LSD1 for the purpose of clarifying molecular functions of LSD1 in HCC. Consequently, we found a total of 392 differentially expressed genes (DEGs) that exhibited highly significant differences in SNU-423 cells, screened out by statistical analysis (*p* < 0.05). Among these, 355 genes were up-regulated after knocking out LSD1, and 37 genes were down-regulated (Figure 4A). In a similar way, a total of 490 significant differentially expressed genes were found in SNU-475 cells. Of these, 274 genes were up-regulated after knocking out LSD1, and 216 genes were down-regulated (Figure 4A). In these two HCC cell lines, we found 65 common significant differentially expressed genes; 57 were up-regulated after knocking out LSD1, and 8 were down-regulated, including LSD1 itself (Figure 4A,B). The MA plot shows the distribution of DEGs in group comparison and indicates significant differences after knocking out LSD1 in both SNU-423 and SNU-475 cell lines (Figure 4C). Then, we conducted gene set enrichment analysis and pathway analysis using Reactome in order to identify the signal pathways regulated by LSD1 in HCC [39]. In this case, we focused on the 64 common significant differentially expressed genes, except for LSD1, in both SNU-423 and SNU-475 HCC cell lines, because we aimed to clarify the common functions of LSD1 in HCC cells, but to explore specific functions of LSD1 in each cell line. Importantly, when we determined that the *p*-value of entities was less than 0.05 as the threshold of this analysis, a limited number of signaling pathways were identified [40]; those were mostly related to the RA pathway, the FGFR and/or Klotho-mediated ligand binding pathway, and the MST-1-mediated signaling pathway (Figure 4D and Supplementary Figures S2–S5). These results imply that LSD1 appears to play a critical role in HCC cells through regulation of the signaling pathways we identified.

## 4. Discussion

In this study, we showed that overexpression of LSD1 is a potential prognostic factor in HCC patients with clinical samples. In addition, we found that LSD1 promotes tumorigenesis and malignancy of HCC in vitro; we identified the signaling pathways regulated by LSD1 in HCC cells.

We previously reported that LSD1 is overexpressed in various types of human cancers and promotes malignant behavior [25,41]. It has also been reported that overexpression of LSD1 is significantly correlates with poor prognosis in prostate cancer, neuroblastoma, and non-small cell lung carcinoma [42,43,44]. In terms of HCC, there has been just one report [45], which shows that overexpression of LSD1 associates with worse 5-year overall survival. However, in reviewing the literature, no data were found on the association between LSD1 expression and DFS or more than five-year over-all survival in HCC patients. In this study, we analyzed the clinical information of 303 HCC patients for more than 5 years—almost 15 years. Patients with positive LSD1 expression had significantly poorer clinical endpoint, not only OS but also DFS rates, than patients with negative LSD1 expression (Figure 1E,F). Moreover, in the multivariate analysis, LSD1 expression had a significant difference on both OS and DFS rates (Table 3 and Table 4). These findings led LSD1 expression to an independent prognostic factor for HCC patients. 

LSD1 is a flavin-dependent monoamine oxidase and affects gene expression by selectively demethylating H3K4me2/me1 and H3K9me2/me1 [23,24,26,46]. These epigenetic changes caused by LSD1 have been shown to play a key role in human tumorigenesis [18,25,47]. However, it is still unclear how LSD1 relates to malignancy of HCC through the epigenetic changes. In this study, we investigated the effects of LSD1 on HCC cells using the Dox-inducible CRISPR/Cas9 system; we established two kinds of LSD1 knock-out cell lines, which are SNU-423-KO cells and SNU-475-KO cells. We first demonstrated colony formation and cell proliferation assays. They showed that LSD1 knockout showed significantly lower number of CFUs and growth rates in both SNU-423 and SNU-475 cell lines compared to the corresponding control cell lines (Figure 2). These findings suggest that LSD1 might play a critical role in proliferation of HCC cells. Additionally, subsequent flow cytometry analysis indicated that LSD1 might also play an important role in cell cycle progression of HCC cells (Figure 3), which is concordant with the results that were previously reported in other types of cancer [25,48].

In the present study, we also conducted RNA-seq analysis to explore molecular mechanisms of how LSD1 is involved in HCC. Our gene set enrichment analysis and pathway analysis using Reactome revealed that LSD1 might mainly regulate the RA pathway in HCC cells. Retinoids have been reported to prevent several kinds of cancers, including HCC; RA coupled with retinoic acid receptor/retinoid X receptor heterodimer exerts its functions by regulating its target genes [49]. Likewise, Cortes et al. recently indicated that retinoic acid receptor beta (RAR-β) was down-regulated in patients with HCC [50], and there is a lot of evidence the RA pathway plays a critical role in hepatocarcinogenesis [49,50,51]. Interestingly, it was reported that the combined effect of RA and LSD1 siRNA had a synergistic effect on promoting the apoptosis of neuroblastoma cells [52], and that also combined treatment with all-*trans* retinoic acid (ATRA), which is the major occurring retinoic acid, and GSK2879552, an LSD1 specific inhibitor, resulted in synergistic effects on enhancing markers of differentiation and promoting cytotoxicity in acute myeloid leukemia across subtypes [53]. Since these findings imply the possibility that a combination therapy of ATRA and some LSD1-specific inhibitors such as Iadademstat might be effective for HCC, we plan to study effects of combined treatment of ATRA and Iadademstat or other LSD1-specific inhibitors on HCC cells. Furthermore, a number of papers have already been published relevant to the involvement of the FGFR and/or Klotho-mediated ligand binding pathway, and the MST1-mediated signaling pathway in HCC [54,55,56,57,58,59]. Taken together, our results imply that LSD1 seems to contribute to hepatocarcinogenesis through regulation of the signaling pathways we identified. On the contrary, there are also some limitations in this study. Firstly, no data regarding chromatin analysis such as ChIP-seq analysis were shown in this study, which means that we are still not sure whether regulation of the signaling pathways identified was directly or indirectly through LSD1-mediated transcriptional regulation. Indeed, we plan to perform comprehensive ChIP-seq analysis to study the status of H3K4me1, H3K4me2, H3K9me1, H3K9me2, and LSD1-binding conditions at the transcriptional regulation of the target genes identified in HCC cells. Secondly, Sehrawat et al. recently reported that LSD1 promoted the survival of prostate cancer cells, including those that are castration-resistant, independently of its demethylase function and of the androgen receptor (AR) [60]. Intriguingly, this demethylase-independent effect was explained in part by activation of a lethal prostate cancer gene network, in collaboration with LSD1’s binding protein, ZNF217 [60]. So far, most of the researchers have focused on the LSD1-demethylase activity, in particular, its transcriptional regulation activity thorough histone demethylation, to clarify functions of LSD1 in human tumorigenesis. However, the aforementioned results reveal that LSD1 is likely to possess other important roles to regulate signal pathways, which are independent of its demethylase activity. Together with the fact that LSD1 can demethylate not only histone proteins, but also non-histone proteins like p53 [18], we need to carefully analyze molecular mechanisms of how LSD1 regulates the signaling pathways we identified.

In conclusion, we found that the histone demethylase LSD1 protein expression in HCC patients was significantly associated with several clinicopathological features, including AFP levels, and that LSD1 protein expression could serve as an independent prognostic factor for HCC patients. Additionally, LSD1 appears to play an important role in growth regulation and cell cycle progression in HCC cells; we also identified the signaling pathways regulated by LSD1 in HCC cells based on the RNA-seq analysis. Further detailed functional analysis may explore the importance of LSD1 as a therapeutic target in hepatocellular carcinoma.

## Figures and Tables

**Table 1:** Clinicopathological characteristics in LSD1 positive and negative groups.

Variables	LSD1-Positive (*n* = 232)	LSD1-Negative (*n* = 71)	*p*-Value
**Patient characteristics**			
Sex, Female	66 (28)	10 (14)	0.018
Male	166 (72)	61 (86)	
Age	69 (62–75)	72 (66–76)	0.11
Alcohol abuse	80 (34)	38 (54)	0.0027
Smoking	90 (39)	34 (48)	0.094
Hepatitis status			
HBV Ag positive	42 (18)	8 (11)	0.39
HCV Ab positive	139 (60)	32 (45)	0.038
No infection	52 (22)	27 (38)	0.013
**Preoperative laboratory tests**			
Albumin, g/dL	4.2 (3.8–4.4)	4.1 (3.8–4.4)	0.78
Total bilirubin, mg/dL	0.8 (0.6–1)	0.7 (0.6–1)	0.16
Prothrombin time, %	87.0 (80.1–96.3)	87.8 (78.3–97.9)	0.63
ICG R15, %	13.0 (9.0–19.0)	12.1 (9.0–16.5)	0.32
AST	45 (30.3–64.8)	37 (25.5–62.5)	0.054
ALT	39 (27.0–64.8)	36 (21.5–55.5)	0.34
Platelet count, ×10^4^/μL	14.1 (10.2–19.0)	16.6 (13.2–19.6)	0.012
Child Pugh grade			1
A	216 (93)	67 (94)	
B	16 (7)	4 (6)	
C	-	-	
AFP, ng/mL	33.8 (6.8–377.1)	7.1 (4.1–29.9)	<0.001
PIVKA-II, mAU/mL	221 (43.0–2333.0)	117 (32.5–875.0)	0.054
CEA, ng/mL	2.1 (1.2–3.0)	1.8 (0.9–2.8)	0.1
CA 19-9, U/mL	10.8 (5.8–19.5)	9.1 (4.2–14.1)	0.017
**Pathological characteristics**			
Tumor maximum size, cm	3.5 (2.5–5.6)	4.0 (2.5–6.1)	0.47
Tumor number, multiple	51 (22)	12 (17)	0.4
HCC differentiation			0.0054
Well	49 (21)	23 (32)	
Moderate	132 (57)	43 (61)	
Poor	51 (22)	5 (7)	
Hepatic vein invasion	43 (19)	5 (7)	0.017
Portal vein invasion	60 (26)	14 (20)	0.34
Tumor category			0.057
T1	30 (13)	8 (11)	
T2	116 (50)	41 (58)	
T3	55 (24)	20 (28)	
T4	31 (13)	2 (3)	
Fibrosis staging F4	119 (51)	23 (32)	0.0061
Activity grading A2-3	72 (31)	18 (25)	0.45
TNM stage (UICC)			0.051
I	32 (14)	7 (10)	
II	114 (49)	42 (59)	
III	54 (23)	20 (28)	
IVA	31 (13)	2 (3)	
IVB	1 (0.4)	-	

Data are *n* (%) for categories, and median (IQR) for continuous data. Abbreviations: AFP, α-fetoprotein; ALT, alanine aminotransferase; AST, aspartate aminotransferase; CA19-9, carbohydrate antigen 19-9; CEA, carcinoembryonic antigen; HBV, hepatitis B virus; HCV, hepatitis C virus; ICG R15, indocyanine green retention test at 15 min; PIVKA-II, protein induced by vitamin K absence or antagonist II.

**Table 2:** Univariate analysis of overall survival (OS) and disease-free survival (DFS) with prognostic factors of HCC.

Variables	Univariate Analysis					
	OS			DFS		
	HR	95% CI	*p*-Value	HR	95% CI	*p*-Value
Sex (male vs. female)	0.89	0.60–1.33	0.57	0.93	0.68–1.28	0.66
Age (≤66 vs. >66)	1.16	0.80–1.66	0.43	0.93	0.70–1.22	0.59
HBV infection (yes or no)	1.09	0.68–1.75	0.72	1.02	0.71–1.48	0.91
HCV infection (yes or no)	1.35	0.94–1.94	0.099	1.23	0.93–1.62	0.14
No infection (yes or no)	0.66	0.43–1.01	0.056	0.83	0.60–1.13	0.23
Child Pugh grade (B or C vs. A)	1.96	1.08–3.55	0.027	1.58	0.93–2.67	0.09
AFP (≤20 vs. >20 ng/mL)	2.35	1.64–3.89	<0.001	1.68	1.28–2.21	<0.001
PIVKA-II (<40 vs. ≥40 mAU/mL)	1.42	0.93–2.17	0.11	1.30	0.94–1.79	0.11
CEA (≤5 vs. >5 ng/mL)	0.81	0.36–1.85	0.62	0.84	0.47–1.50	0.55
CA19-9 (≤37 vs. >37 U/mL)	1.20	0.61–2.37	0.6	0.87	0.48–1.56	0.64
Tumor size (≤5 vs. >5 cm)	1.45	1.01–2.10	0.047	1.42	1.06–1.91	0.018
Tumor number (multiple vs. single)	2.14	1.46–3.14	<0.001	2.39	1.75–3.26	<0.001
Differentiation (mod/por vs. wel)	1.57	1.01–2.43	0.043	1.48	1.06–2.07	0.021
Hepatic vein invasion (yes vs. no)	2.36	1.56–3.55	<0.001	2.40	1.70–3.37	<0.001
Portal vein invasion (yes vs. no)	2.13	1.48–3.08	<0.001	1.93	1.43–2.60	<0.001
Tumor category (T3–4 vs. 1–2)	3.00	2.12–4.25	<0.001	2.44	1.85–3.22	<0.001
Fibrosis stage (4 vs. 0–3)	1.59	1.12–2.26	0.0099	1.61	1.23–2.12	<0.001
Activity stage (2–3 vs. 0–1)	1.01	0.69–1.48	0.95	1.18	0.88–1.54	0.27
TNM stage (III–IV vs. I–II)	2.97	2.09–4.22	<0.001	2.57	1.95–3.39	<0.001
LSD1 (positive vs. negative)	2.16	1.31–3.56	0.0024	1.75	1.24–2.48	0.0016

**Table 3:** Multivariate analysis of OS and DFS with prognostic factors of HCC.

Variables	Multivariate Analysis					
	OS			DFS		
	HR	95% CI	*p*-Value	HR	95% CI	*p*-Value
HCV infection (yes or no)	1.09	0.70–1.69	0.70	1.06	0.76–1.48	0.74
Child Pugh grade (B or C vs. A)	1.53	0.71–3.30	0.27	1.65	0.88–3.08	0.17
AFP (≤20 vs. >20 ng/mL)	1.66	1.07–2.57	0.0025	1.28	0.91–1.79	0.15
PIVKA-II (<40 vs. ≥40 mAU/mL)	1.178	0.71–1.96	0.53	1.07	0.74–1.55	0.73
Tumor size (≤5 vs. >5 cm)	1.08	0.65–1.80	0.76	1.06	0.71–1.59	0.78
Tumor number (multiple vs. single)	1.20	0.71–2.01	0.49	1.33	0.85–2.07	0.21
Differentiation (mod/por vs. wel)	1.07	0.62–1.85	0.80	0.71	0.45–1.13	0.15
Hepatic vein invasion (yes vs. no)	1.02	0.57–1.81	0.96	1.14	0.70–1.83	0.60
Portal vein invasion (yes vs. no)	1.18	0.72–1.93	0.52	1.24	0.85–1.81	0.27
Tumor category (T3–4 vs. 1–2)	2.26	0.37–13.8	0.38	0.56	0.13–2.33	0.42
Fibrosis stage (4 vs. 0–3)	1.23	0.65–2.51	0.48	1.43	0.82–2.51	0.21
TNM stage (III–IV vs. I–II)	0.96	0.16–5.87	0.97	3.51	0.84–14.7	0.086
LSD1 (positive vs. negative)	1.98	1.07–3.64	0.029	1.74	1.15–2.64	0.0086

**Table 4:** Multivariate analysis of OS and DFS with prognostic factors of HCC.

Variables		Multivariate Analysis		
		HR	95% CI	*p*-Value
**OS**	AFP (≤20 vs. >20 ng/mL)	1.76	1.18–2.61	0.005
	Tumor category (T3–4 vs. 1–2)	3.01	2.05–4.43	<0.001
	LSD1 (positive vs. negative)	2.18	1.22–3.92	0.009
**DFS**	Fibrosis stage (4 vs. 0–3)	1.70	1.06–2.70	0.0026
	TNM stage (III–IV vs. I–II)	2.22	1.52–3.24	<0.001
	LSD1 (positive vs. negative)	1.67	1.12–2.48	0.012

## Supplementary Materials

The following are available online at https://www.mdpi.com/2218-273X/9/12/810/s1, Figure S1: Effects of Dox on the growth of SNU423 and SNU475 HCC cells., Figure S2: RA biosynthesis pathway, Figure S3: Signaling by Retinoic Acid., Figure S4: FGFR1c and Klotho ligand binding and activation., Figure S5: Signaling by MST1., Table S1: Information of certificated cell lines., Table S2: RNA-seq QC results.
