# Supplementary material for: Deregulation of the Histone Lysine-Specific Demethylase 1 Is Involved in Human Hepatocellular Carcinoma
Source: Biomolecules. 2019 Dec 1;9(12):810. doi: 10.3390/biom9120810 (PMC6995592; doi:10.3390/biom9120810)
Supplement: Supplementary file 1 [file biomolecules-09-00810-s001.pdf]

Supplementary information

The file contains

Figures S1-S5

Tables S1-S2

References

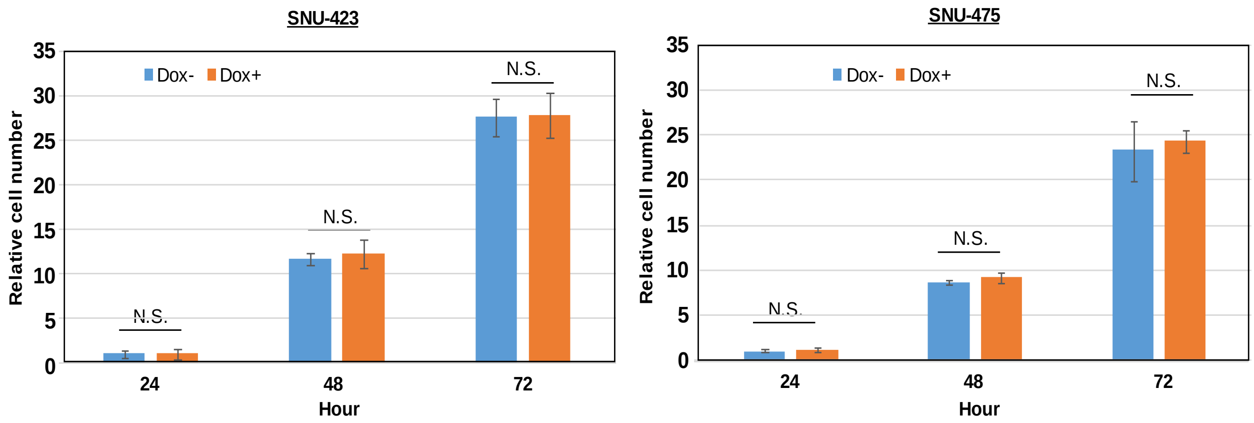

Figure S1. Effects of Dox on the growth of SNU-423 and SNU-475 HCC cells. Cell proliferation assays were performed to determine the cell viability of HCC cells with CCK-8 kit after 1 µg/mL of Dox treatment for 24 h. X-axis shows time course (hour) after replacing medium without Dox. Relative cell number means that the cell number of Dox- at 24 hours is 1 in both SNU-423 cells and SNU-475 cells. All date are represented as means±s.d. N.S.; not significant.

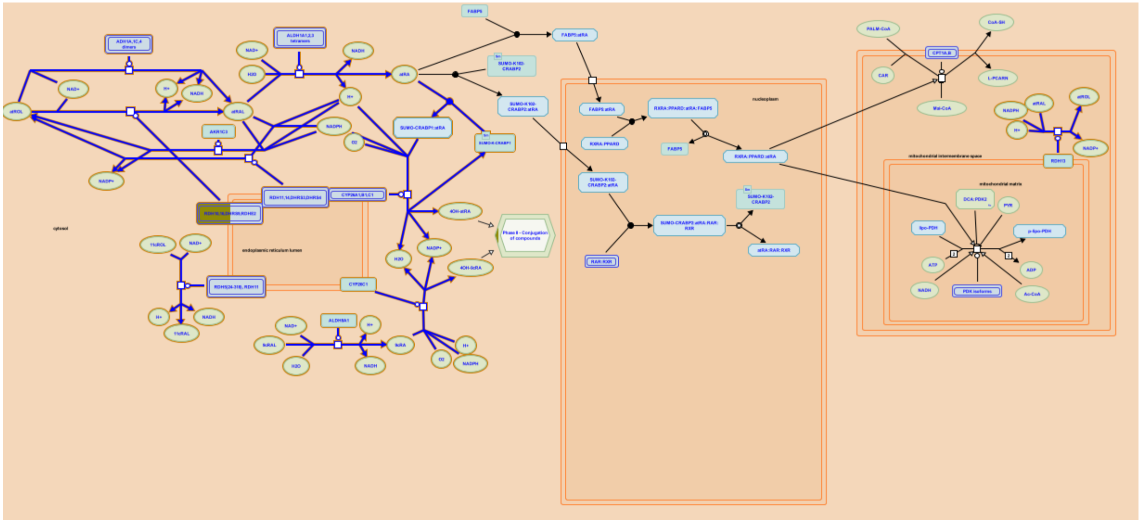

Figure S2. RA biosynthesis pathway (Reactome Stable ID: R-HSA-5365859, https://reactome.org/content/detail/R-HSA-5365859). The major activated retinoid, all-trans-retinoic acid (atRA) is produced by the dehydrogenation of all-trans-retinol (atROL) by members of the short chain dehydrogenase/reductase (SDR) and aldehyde dehydrogenase (RALDH) gene families [1,2].

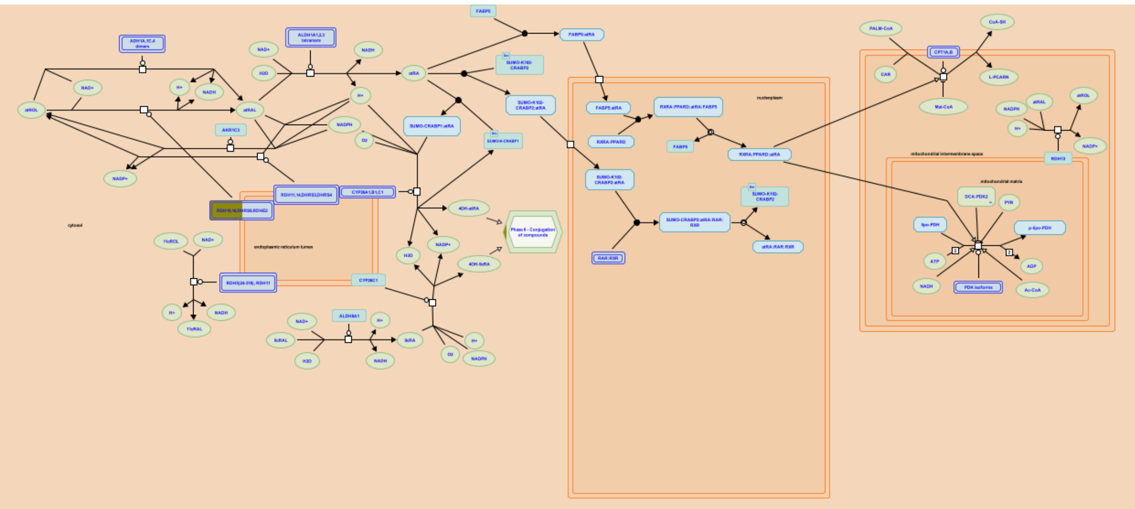

Figure S3. Signaling by Retinoic Acid (Reactome Stable ID: R-HSA-5362517, https://reactome.org/content/detail/R-HSA-5362517). Vitamin A (retinol) can be metabolised into active retinoid metabolites that function either as a chromophore in vision or in regulating gene expression transcriptionally and post-transcriptionally. Genes regulated by retinoids are essential for reproduction, embryonic development, growth, and multiple processes in the adult, including energy balance, neurogenesis, and the immune response. The retinoid used as a cofactor in the visual cycle is 11-cis-retinal (11cRAL). The non-visual cycle effects of retinol are mediated by retinoic acid (RA), generated by two-step conversion from retinol [2]. All-trans-retinoic acid (atRA) is the major activated metabolite of retinol. An isomer, 9-cis-retinoic acid (9cRA) has biological activity, but has not been detected in vivo, except in the pancreas. An alternative route involves BCO1 cleavage of carotenoids into retinal, which is then reduced into retinol in the intestine [3]. The two isomers of RA serve as ligands for retinoic acid receptors (RAR) that regulate gene expression [1]. RA is catabolised to oxidised metabolites such as 4-hydroxy-, 18-hydroxy- or 4-oxo-RA by CYP family enzymes, these metabolites then becoming substrates for Phase II conjugation enzymes [4].

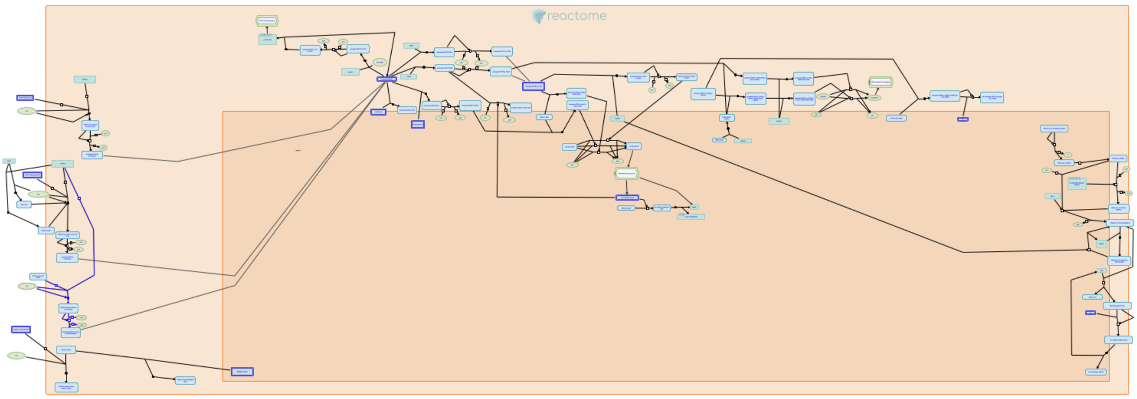

Figure S4. FGFR1c and Klotho ligand binding and activation (Reactome Stable ID: R-HSA-190374 https://reactome.org/content/detail/R-HSA-190374). FGF23 is a member of the endocrine subfamily of FGFs. It is produced in bone tissue and regulates kidney functions. Klotho is essential for endogenous FGF23 function as it converts FGFR1c into a specific FGF23 receptor [5,6].

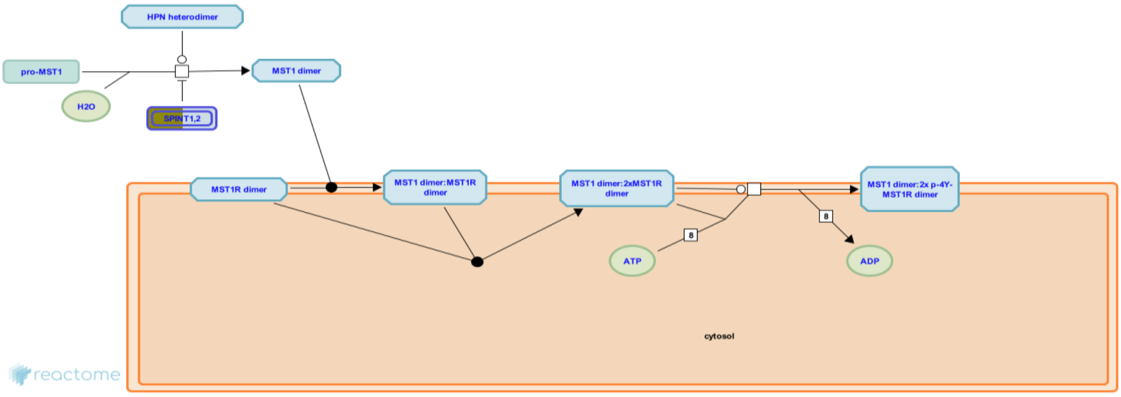

Figure S5. Signaling by MST1 (Reactome Stable ID: R-HSA-8852405, https://reactome.org/content/detail/R-HSA-8852405). Inflammatory mediators such as growth factors produced by macrophages play an important role in the inflammatory response occurring during bacterial infection, tissue injury and immune responses. Many growth factors and their receptor-type protein tyrosine kinases (RTKs) play a critical role in inflammation, wound healing and tissue remodelling. The growth factor hepatocyte growth factor-like protein (MST1, also known as macrophage-stimulating protein, MSP) binds to a specific receptor, macrophage-stimulating protein receptor (MST1R, also known as RON, recepteur d'origine nantais). MST1 belongs to the kringle protein family, which includes HGF and plasminogen. It is produced by the liver and circulates in the blood as a biologically-inactive single chain precursor (pro-MST1). Proteolytic cleavage of pro-MST1 into the biologically-active MST1 dimer is necessary for receptor binding. Cleavage occurs during blood coagulation and at inflammatory sites, the resultant MST1 dimer then binds MST1R receptors on local macrophages. MST1R is ubiquitously expressed but mainly in epithelial cells.MST1 binding to MST1R promotes receptor homodimerisation which in turn allows autophosphorylation of two tyrosine residues within the catalytic site which regulates kinase activity and allows phosphorylation of the carboxy-terminal binding site of the receptor. The docking site is essential for downstream signaling through direct and indirect binding of SH2 domain-containing adaptor proteins such as GRB2, PI3K, and SRC. MST1/MST1R signaling plays a dual role in regulating inflammation; initially stimulating chemotaxis and phagocytosis (macrophage activation) and then exerts broad inhibitory effects on macrophages, limiting the extent of inflammtory responses [7]. MST1R is upregulated in many epithelial cancers where it is thought to play a role in the progression of these types of cancer [8].

Table S1. Information of certificated cell lines.

Name	Origin	Certification institution	Tested method	DNA profile or characteristics
SNU-423	human hepatocellular carcinoma	ATCC	STR	Amelogenin: X,Y CSF1PO: 11,12 D13S317: 10,13 D16S539: 9 D5S818: 10 D7S820: 12 THO1: 6,9 TPOX: 8 vWA: 15
SNU-475	human hepatocellular carcinoma	ATCC	STR	Amelogenin: X,Y CSF1PO: 11,12 D13S317: 8,11 D16S539: 12 D5S818: 10,13 D7S820: 7,12 THO1: 7,9 TPOX: 8,9 vWA: 14

ATCC; American Type Culture Collection

Table S2. RNA-seq QC results.

Sample Name	Biological replicates	sgRNA clone#	Clean Reads	Clean bases	Read length (bp)	Q20(%)	GC(%)
SNU-423-LSD1KO-Dox-	1	1	51,537,846	5,153,784,600	100	97.12%	49.39%
SNU-423-LSD1KO-Dox-	2	1	51,660,490	5,166,049,000	100	97.18%	49.24%
SNU-423-LSD1KO-Dox-	3	1	51,592,146	5,159,214,600	100	96.93%	48.90%
SNU-423-LSD1KO-Dox-	4	1	51,721,568	5,172,156,800	100	97.15%	48.61%
SNU-423-LSD1KO-Dox+	1	1	51,516,906	5,151,690,600	100	96.78%	49.21%
SNU-423-LSD1KO-Dox+	2	1	51,974,118	5,197,411,800	100	96.57%	48.59%
SNU-423-LSD1KO-Dox+	3	1	47,785,494	4,778,549,400	100	96.99%	48.86%
SNU-423-LSD1KO-Dox+	4	1	51,751,432	5,175,143,200	100	96.96%	48.80%
SNU-475-LSD1KO-Dox-	1	2	51,188,246	5,118,824,600	100	96.86%	48.68%
SNU-475-LSD1KO-Dox-	2	2	47,684,054	4,768,405,400	100	96.71%	48.96%
SNU-475-LSD1KO-Dox-	3	2	51,621,900	5,162,190,000	100	96.71%	48.79%
SNU-475-LSD1KO-Dox-	4	2	51,433,440	5,143,344,000	100	96.64%	49.16%
SNU-475-LSD1KO-Dox+	1	2	51,792,872	5,179,287,200	100	96.87%	48.99%
SNU-475-LSD1KO-Dox+	2	2	51,143,418	5,114,341,800	100	97.10%	48.79%
SNU-475-LSD1KO-Dox+	3	2	51,677,446	5,167,744,600	100	96.81%	49.10%
SNU-475-LSD1KO-Dox+	4	2	47,218,394	4,721,839,400	100	96.76%	49.03%

References

1. Das, B.C.; Thapa, P.; Karki, R.; Das, S.; Mahapatra, S.; Liu, T.C.; Torregroza, I.; Wallace, D.P.; Kambhampati, S.; Van Veldhuizen, P., et al. Retinoic acid signaling pathways in development and diseases. *Bioorg Med Chem* **2014**, *22*, 673-683.
2. Napoli, J.L. Physiological insights into all-trans-retinoic acid biosynthesis. *Biochim Biophys Acta* **2012**, *1821*, 152-167.
3. Harrison, E.H. Mechanisms involved in the intestinal absorption of dietary vitamin A and provitamin A carotenoids. *Biochim Biophys Acta* **2012**, *1821*, 70-77.
4. Ross, A.C.; Zolfaghari, R. Cytochrome P450s in the regulation of cellular retinoic acid metabolism. *Annu Rev Nutr* **2011**, *31*, 65-87.
5. Kurosu, H.; Ogawa, Y.; Miyoshi, M.; Yamamoto, M.; Nandi, A.; Rosenblatt, K.P.; Baum, M.G.; Schiavi, S.; Hu, M.C.; Moe, O.W., et al. Regulation of fibroblast growth factor-23 signaling by klotho. *J Biol Chem* **2006**, *281*, 6120-6123.
6. Urakawa, I.; Yamazaki, Y.; Shimada, T.; Iijima, K.; Hasegawa, H.; Okawa, K.; Fujita, T.; Fukumoto, S.; Yamashita, T. Klotho converts canonical FGF receptor into a specific receptor for FGF23. *Nature* **2006**, *444*, 770-774.
7. Wang, M.H.; Zhou, Y.Q.; Chen, Y.Q. Macrophage-stimulating protein and RON receptor tyrosine kinase: potential regulators of macrophage inflammatory activities. *Scand J Immunol* **2002**, *56*, 545-553.
8. Kretschmann, K.L.; Eyob, H.; Buys, S.S.; Welm, A.L. The macrophage stimulating protein/Ron pathway as a potential therapeutic target to impede multiple mechanisms involved in breast cancer progression. *Curr Drug Targets* **2010**, *11*, 1157-1168.
